# Beyond the Blood:Brain Barrier: The Importance of Central Nervous System (CNS) Pharmacokinetics for the Treatment of CNS Tumors, Including Diffuse Intrinsic Pontine Glioma

**DOI:** 10.3389/fonc.2018.00239

**Published:** 2018-07-03

**Authors:** Katherine Elizabeth Warren

**Affiliations:** Neuro-Oncology Section, Pediatric Oncology Branch, National Cancer Institute (NCI), Rockville, MD, United States

**Keywords:** central nervous system pharmacokinetics, blood:brain barrier, brain tumor, diffuse intrinsic pontine glioma, drug delivery

## Abstract

Over the past decade, we have made considerable progress in establishing diffuse intrinsic pontine glioma (DIPG) as a disease entity and developing preclinical tools to interrogate potential therapeutics. However, translation to improved clinical outcomes in children with DIPG has not yet been realized. This is in part due to difficulties encountered in delivering active drugs adequately to the tumor site. However, most preclinical evaluations gloss over the fundamental concepts of central nervous system (CNS) pharmacokinetics and requirements needed to optimize drug delivery and exposure and translate this into efficacious therapy. This article discusses not only the blood:brain barriers but additional barriers to drug delivery for CNS tumors and pharmacokinetic principles that need to be addressed and considered.

## Introduction

A critical determinant of drug efficacy is achieving adequate exposure of an active agent, in its unbound (free) state, at its site of action. Failure to do so has been identified as *the* major obstacle in successful treatment of many tumors of the central nervous system (CNS) given that effective therapies have been identified *in vitro* but yet not successfully translated in patients. This is particularly true for children with diffuse intrinsic pontine glioma (DIPG) (1–6). We now have tools to preclinically identify and evaluate potential therapeutics in DIPG disease-specific cell lines and animal models (7, 8). Information that is critical to successfully translate preclinical activity of agents into the clinic for children with DIPG must include delivery of the agent to the tumor site, yet these studies are infrequently performed prior to evaluating agents in clinical trials. When preclinical studies are performed, they are often limited to evaluation of drug concentration at a single time point within CNS tissue or tumor. Most agents are unable to sufficiently enter the brain parenchyma or tumor tissue to exert adequate antitumor effects (9). Restricted drug delivery to the CNS is most frequently attributed to the blood:brain barrier (BBB), yet additional factors which hamper drug delivery and distribution within the CNS must also be recognized and addressed, particularly now that alternate drug delivery techniques that bypass the BBB are clinically feasible. Application of CNS pharmacokinetics is critical to rationally design new drugs and therapeutic trials with the aim of optimizing therapeutic exposure at the tumor site. It is also vital to understand CNS pharmacokinetics when considering the risk:benefit ratio of hyped treatments with unrealistic promise offered to desperate families of children with DIPG. Yet, our understanding of CNS pharmacokinetics in brain parenchyma and extracellular space (ECS), and its relationship to the cerebrospinal fluid (CSF) and drug distribution is incomplete. This review will encompass our current understanding of CNS pharmacokinetics including drug delivery and distribution within the CNS and the effects of alternate administration techniques.

## Drug Entry into the CNS

Requirements for drug efficacy (Table 1) include adequate exposure of an agent at its site of action. For CNS tumors, this generally implies delivery of systemically administered agents into the CNS, and distribution within the CNS parenchyma to the tumor site. This first hurdle, i.e., crossing into the CNS, has garnished most attention. Drugs administered systemically must pass from the blood circulation into the CNS by crossing a barrier whose fundamental function is to protect the CNS from harmful substances while concurrently supplying CNS tissue with needed nutrients and eliminating CNS waste products (10). Delivery of drugs to the CNS is impeded by this BBB primarily due to tight junctions between the endothelial cells, unavailability of transport vesicles, and lack of transcellular pathways especially for hydrophilic drugs (11). Most research focuses on the BBB as the major obstacle to adequate drug delivery to CNS tumors, yet similar issues apply to the blood:tumor barrier (BTB) and blood:CSF barrier (BCSFB); other factors, such as abnormal intratumoral vasculature, increased interstitial pressure, and peritumoral edema, also hinder drug delivery to tumors in the CNS.

**Table 1:** Requirements for anti-tumor drug efficacy.

Tumor cells must be sensitive (i.e., active drug) Drug must be delivered to its site of action (e.g., tumor cells) Drug must be present at the tumor site in its active, unbound form Adequate exposure (i.e., effective concentrations for a long enough period of time) at the active (tumor) site Patient must be able to tolerate the dose and schedule necessary to achieve above If targeted agent, target must be present

## The Blood:Brain Barrier

**Figure 1:**
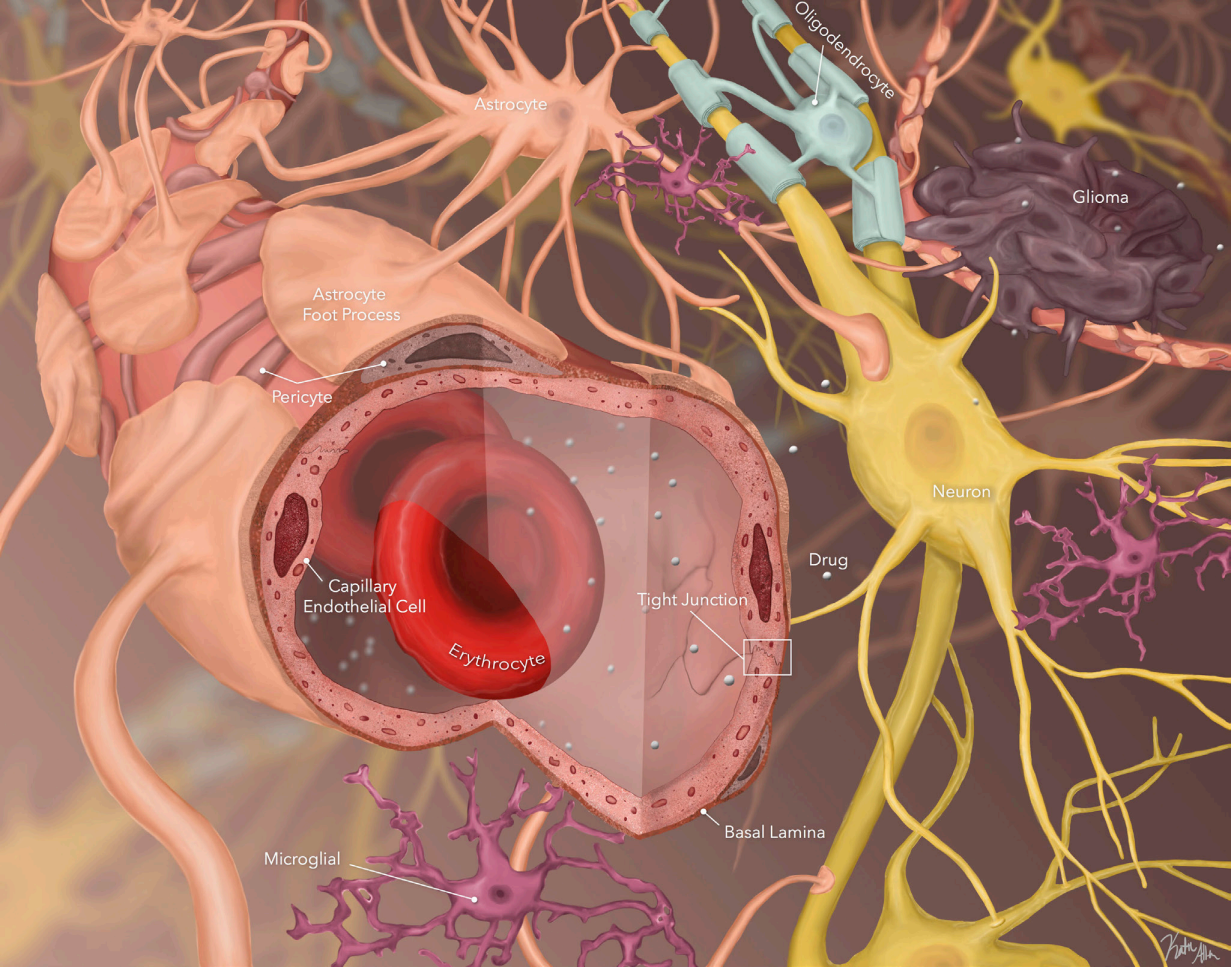
Schematic of blood:brain barrier (BBB), brain parenchyma, and stroma. Small, unbound, lipophilic agents are able to cross the BBB. They then must cross through brain parenchyma by diffusion across the extracellular space to reach tumor cells (*image by Katie Allen*).

The blood:brain barrier (Figure 1) is a neurovascular unit composed of specialized highly polarized endothelial cells, their surrounding pericytes, astrocytic foot processes, neurons, mast cells, microglia, and circulating immune cells (12, 13). At the molecular level, the BBB comprises tight junction proteins, adherence proteins, transporters, basal lamina, and extracellular matrix. The tight junctions and adherens proteins prevent paracellular diffusion (i.e., movement between cells), so drugs entering the brain parenchyma generally must cross the luminal and abluminal plasma membranes of the endothelial cell. While most small lipophilic substances may cross the BBB by simple diffusion, other potential processes include facilitated (carrier-mediated) diffusion, simple diffusion through an aqueous channel, active transport, or paracellular diffusion. Factors that determine how much drug crosses the BBB (Table 2) include physicochemical properties of the agent, e.g., lipid solubility, hydrogen bonding, molecular mass, as well as cerebral blood flow, metabolism, degradation, and clearance of drug in the bloodstream (high systemic clearance limits drug availability for CNS penetration) and protein binding (only free, i.e., unbound, drug is available to cross the BBB *via* transendothelial diffusion) (12). Drug characteristics that are favorable for crossing the BBB are therefore high lipophilicity, small size and molecular weight, and low hydrogen-bonding potential (i.e., the drug is unionized at physiologic pH).

**Table 2:** Determinants of central nervous system penetration.

Physicochemical properties of the drug
Size Lipophilicity Degree of hydrogen bonding Molecular mass P-glycoprotein (MDR1 or ABCB1) substrate
Cerebral blood flow
Drug metabolism, degradation, and clearance
Degree of protein binding
Integrity of the blood:brain barrier

Importantly, *the BBB is not static*, but rather dynamic, interacting with its microenvironment and responding to the needs of the CNS (12, 13). Its permeability is, at least in part, controlled by intra- and intercellular signaling among the endothelial cells and surrounding astrocytes and neurons (14). Specific transporters allow necessary water-soluble molecules, such as glucose, to rapidly traverse the BBB and mediate transport of large molecules (e.g., some proteins). The affinity of drug for its carrier/transporter is a critical determinant of transport into or out of CNS for those agents requiring specific transporters. Active transport also requires energy and allows transport of drugs against a concentration gradient. Uptake/influx transporters, which facilitate entry *into* the brain, include organic anion transporting polypeptides, nucleoside transporters, monocarboxylate transporters, and peptide transport systems (15). Active *efflux* transporters are also present at the BBB and serve to restrict entry of many chemotherapeutic agents into the CNS. Efflux transporters include P-glycoprotein (Pgp), breast cancer resistance proteins, and multidrug resistance proteins. There is an association between polarity and active efflux at the BBB, with increased interaction of drug efflux transporter protein with agents that are able to form a greater number of hydrogen bonds (16).

**Figure 2:**
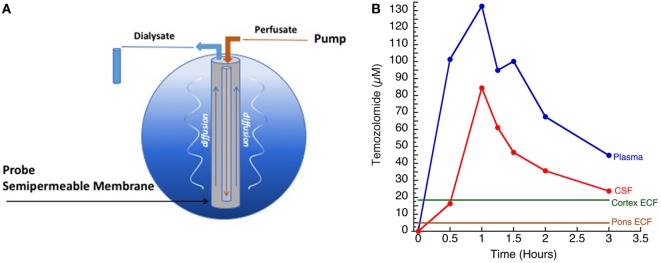
**(A)** Illustration of microdialysis setup. Drug of interest (TMZ) was administered systemically. **(B)** Concentration versus time curves of temozolomide measured in plasma, cerebrospinal fluid (CSF), cortex ECF, and pontine ECF (*courtesy of Cindy McCully*).

In addition, *the BBB is not homogeneous*, having different expression of active efflux and influx transporters and spatial differences, which result in variable penetration of drugs across distinct areas of the CNS. Although data on geographic variability of CNS drug penetration are limited, initial studies suggest this may have significant consequences for the treatment of CNS tumors, particularly DIPG. Despite numerous clinical trials, no chemotherapeutic agent has ever demonstrated significant efficacy against DIPG in a clinical trial, and radiation therapy remains the primary treatment modality (4, 17–21). Data suggest that the pons has a super-BBB, further restricting entry of substances into the brainstem parenchyma compared with other areas in the brain, which fits given that areas in the pons control basic life functions. Using a non-human primate model, we performed *in vivo* microdialysis (MD) studies (Figure 2), and simultaneously compared the concentration of the alkylating agent, temozolomide, in the extracellular fluid of the cortex and pons, as well as in plasma and CSF, after intravenous administration. This study consistently demonstrated significantly lower levels of temozolomide in the pons compared with the cortex and CSF, suggesting more limited penetration in the pons (22, 23). This heterogeneous drug penetration into the CNS and the apparent “super-BBB” that exists to significantly limit penetration in the pons has critical implications for preclinical drug penetration studies and treatment of CNS tumors; one cannot assume drug exposure is the same at different locations in the brain, and therefore assuring adequate exposure at the active site must consider the location being assessed and the location being targeted. In addition, there is both intra- and inter-patient variability in enhancement patterns, which may be disease subtype related (1). Some patients demonstrate limited or no enhancement at diagnosis, while others may have significant nodular or cystic enhancement. In all cases, enhancement on a post-contrast MRI is not representative of the entire tumor burden (24).

Interestingly, this BBB is absent in the circumventricular organs, which are small anatomically defined vascular regions that include the area postrema, lamina terminalis, subfornical organ, subcommissural organ, posterior pituitary, median eminence, and the pineal gland. These are extensively vascular areas where the capillaries are fenestrated. How these areas effect and regulate drug penetration into the CNS is incompletely understood but are active areas of study particularly as they may be a gateway for trafficking of immune cells.

## The Blood:Tumor Barrier

The physical and biochemical integrity of the BBB is frequently disrupted in CNS tumors. This is classically recognized by enhancement on magnetic resonance imaging after administration of a gadolinium-based contrast agent, with the hydrophilic contrast agent leaking out of the vascular lumen and into the ECS in areas with a disrupted BBB. However, breakdown of the BBB within the tumor is almost never homogeneous and does not represent the entire extent of the tumor. This is particularly true for malignant gliomas, where the tumor edge, which contains infiltrating tumor cells, frequently does not enhance. Invasive malignant glioma tumor cells have been found in normal appearing brain (25); in addition, tumor recurrence has occurred in the contralateral hemisphere even after removal of the entire ipsilateral hemisphere (26) demonstrating wide infiltration of tumor cells not detected by contrast enhancement and in areas presumably having an intact BBB. In areas that do enhance, tumor vasculature is often abnormal, with areas of dilatation and poor flow. Dysfunction of this BTB may be inferred by vasogenic edema detected on MRI (27), with resulting abnormal accumulation of fluid in the brain parenchyma creating increased intratumoral and peritumoral pressure.

From a treatment perspective, BTB dysfunction can also affect drug distribution; the degree of BTB dysfunction differs within different areas of a tumor in an individual patient and also from patient to patient (27). As a result, contrast enhancement alone should not be used as a determinant of adequate drug delivery to a tumor bed. With our limited understanding of the BTB, contrast enhancement is a poor prognostic or predictive biomarker of chemotherapy response (and assumption of BBB breakdown). For example, we know that vincristine and carboplatin have limited CNS penetration, yet pediatric patients with non-enhancing low-grade gliomas may respond to therapy. By contrast, adults with glioblastoma multiforme treated with surgical resection, radiation therapy, and temozolomide most frequently fail within the radiation volume which is typically centered on the area of contrast enhancement (27). How do we then rationalize the fact that non-enhancing tumors commonly respond to systemically administered chemotherapeutic agents and some enhancing tumors do not? Certainly, other factors in addition to BBB breakdown are at play.

**Figure 3:**
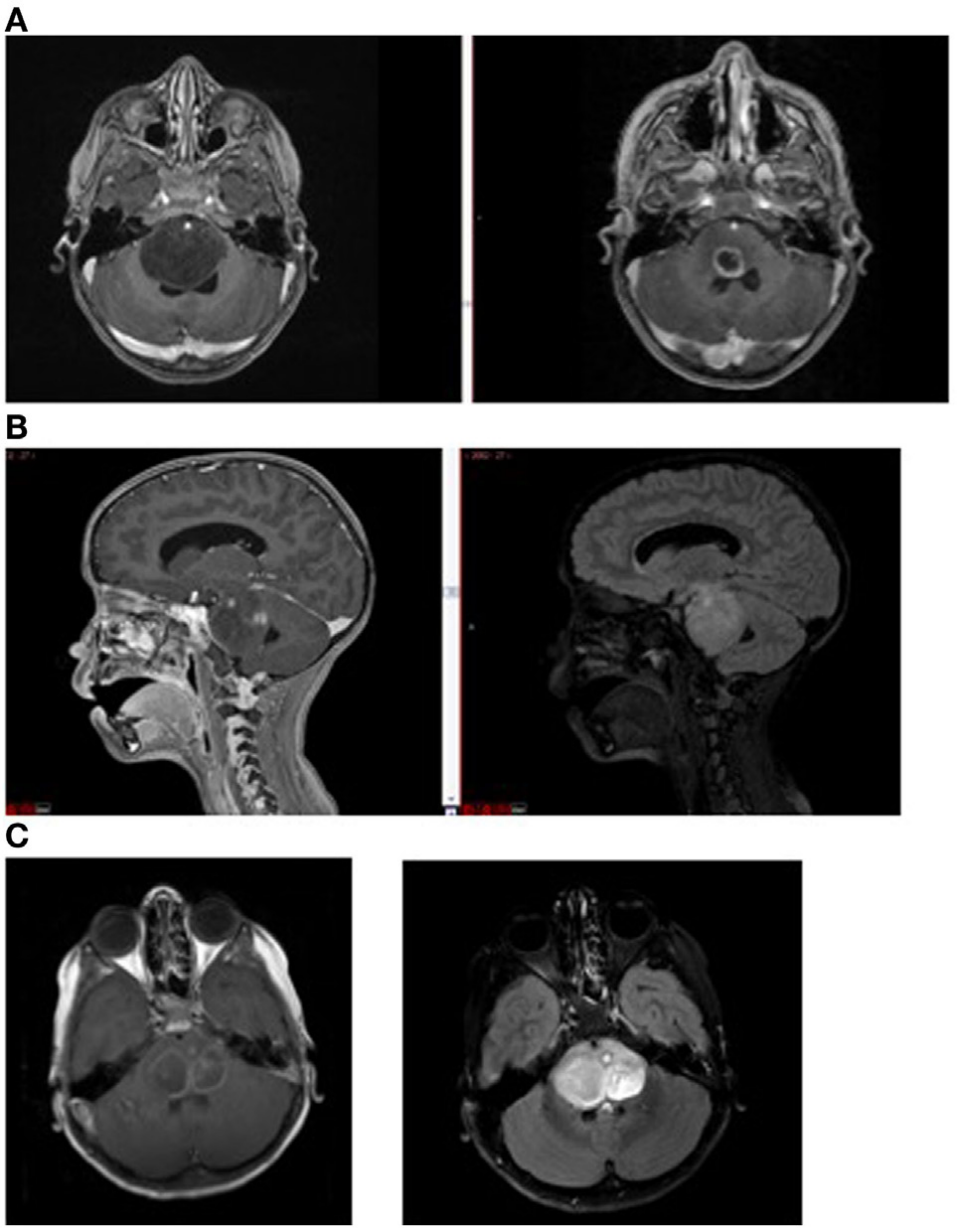
Magnetic resonance imaging demonstrating **(A)** non-enhancing tumor at diagnosis (left) and enhancement (right) following radiation therapy; **(B)** tumor necrosis (enhancement at diagnosis, left) and FLAIR image (right) showing extent of tumor beyond area of enhancement, and **(C)** intratumoral cysts. Areas of contrast enhancement do not represent tumor burden in diffuse intrinsic pontine glioma and do not ensure adequate drug delivery.

In children with DIPG, tumors frequently do not enhance significantly at diagnosis, implying the BBB is intact over much of the tumor and supporting the idea of a super-BBB (Figure 3). Areas that do enhance at diagnosis are frequently near the center of the tumor, implying tumor necrosis from rapid growth, hypoxia, and inability to achieve necessary nutrients. Following radiation therapy, increased enhancement is observed, likely representing BBB breakdown from the effects of radiation. Not infrequently, cysts with enhancing cyst walls are present or develop after radiation therapy. While these represent common findings of dysfunctional BBB, this is not sufficient to improve drug delivery significantly enough to be effective.

## The Blood:CSF Barrier

In contrast to the BBB and BTB, BCSFB is formed by modified epithelial cells rather than endothelial cells. This BCSFB is located at the choroid plexus and the arachnoid membrane (28). The choroid plexus, composed in part by a cuboidal epithelial barrier with fenestrated vasculature, is located in the ventricular systems and secretes CSF, which occupies the ventricular systems and the subarachnoid space, and has extensive two-way communication with the CNS interstitial fluid. The choroid plexus is important in CNS homeostasis because it aids in controlling the composition of CSF and brain interstitial fluid (28). Most waste products from the interstitial fluid are transported to the CSF. Exchange between blood and CSF at the choroid plexus is affected by local blood flow, fenestrated capillaries, and the substantial surface area created by membrane folding and microvilli (28).

The pathway of CSF flow is an important determinant of drug exposure at different sites within the CNS. From the ventricles, CSF circulates into the cisternal and subarachnoid spaces, which are characterized by the leptomeninges containing blood vessels. The CSF and perivascular (Virchow–Robin) spaces surrounding the vessels penetrate the brain parenchyma and allow fluid exchange between CSF and perivascular fluid (28, 29). The CSF has particular characteristics that play key roles in drug delivery throughout the leptomeninges and can affect drug delivery from the CSF into the CNS tissue: (a) although CSF flows, substances are not homogeneously mixed throughout. Drugs that are administered into the CSF circulate inhomogeneously; this circulation can be affected by gravity, presence of increased proteins or tumor, e.g., leptomeningeal disease, or presence of a ventriculo-peritoenal shunt; (b) CSF is continuously produced, therefore, CSF turns over and substances are “diluted out” by continuous CSF production; (c) some drugs may be metabolized or eliminated from CSF into bloodstream or glymphatic system.

## Drug Distribution within the CNS

To date, administration techniques that bypass the BBB or disrupt the BBB have not demonstrated improved outcome for patients with CNS tumors, including those with DIPG. This may be due to a further major obstacle to drug delivery, i.e., traversing the brain parenchyma and stroma to reach the site of action (tumor cells) (Figure 1). To be efficacious, the drug not only has to cross the BBB but be delivered to the tumor site, and be present at the tumor site, in its active form, in effective concentrations for an appropriate time period. Several studies have demonstrated that drug concentrations drop off significantly across the brain parenchyma from the site of infusion or site of entry in the CNS. Few studies address the actual drug concentration at the tumor site, and fewer still address concentration over time at the active site, yet these are critical determinants of therapeutic efficacy.

Once across the CNS barriers (i.e., BBB, BTB, and BCSFB), substances must navigate the surrounding astrocytes, pericytes, neurons, and microglia through the ECS. Importantly, for those small lipophilic substances that were successful in transcellular diffusion, this requires partitioning from the lipid environment of the BBB endothelial cell membrane back into the aqueous environment of the interstitial fluid (12). For agents crossing the BCSFB, the charge of the drug influences partitioning between the aqueous and lipid compartments as the CSF is normally more acidic than plasma (pH of 7.33 compared with 7.4 in plasma) (30).

The ECS in the CNS is the fluid-filled channel between cells that is critical for neuronal function, allows for intercellular communication, and, importantly, represents the conduit for drug distribution and delivery in the CNS. It is a complex microenvironment, with a measured width of only ~40 nm between cells but normally occupying approximately 20% of brain tissue (31). The ECS includes interstitial spaces filled with extracellular fluid, as well as blood vessels, perivascular space, and the ventricular and subarachnoid spaces. The extracellular fluid in the interstitial space (i.e., interstitial fluid) is similar to CSF with added extracellular matrix molecules, such as proteoglycans and hyaluronan, which can impede drug diffusion (31). Extracellular matrix is composed of the negatively charged glycosoaminoglycans and proteoglycans, which increase viscosity of the ECS, influence diffusion of cations and anions due to its inherent negative charges, and may regulate the width of the ECS through hydration of hyaluronan (32). It is estimated that the effective or apparent diffusion coefficient for small molecules in the ECS is 40% of that in free solution primarily due to geometrical constraints (which create an increased diffusion path length around cells), dead space, obstruction from extracellular matrix molecules, and extracellular matrix charge which can inhibit diffusion of charged molecules (31).

Drug distribution within the CNS depends on several factors including normal physiological fluid movement (cerebral blood flow, CSF flow, and extracellular fluid movement), exchange between the extracellular and intracellular compartments, and pH. Most small molecules distribute in the interstitial fluid by the process of diffusion, moving down their concentration gradient; the degree of movement along the concentration gradient depends on the drug’s molecular size, other molecular properties, temperature, and pressure. Diffusion in the ECS can be affected by the extracellular matrix, edema, ischemia, osmolarity, and cellularity (31). As has been demonstrated, diffusion across the brain parenchyma is limited to just a few millimeters beyond the site of infusion (33).

The structure and content of the ECS are the primary determinants of molecular movement through brain tissue (32). Notably, the ECS is not homogeneous throughout the CNS and diffusion rates are not the same in every direction, i.e., they are anisotropic. For example, substances diffuse more readily along a fiber tract or axon than across it (32), a characteristic that can be measured with diffusion tensor imaging. A number of factors, including many associated with CNS tumors, can affect diffusion, including

- ischemia, which creates transmembrane ionic shifts, water redistribution (from extracellular to intracellular), causing expansion of neurons and glia, and altering ECS (32),
- gliosis, which is associated with astrocytic hyperplasia and hypertrophy, resulting in a decreased volume fraction of the ECS,
- hydrocephalus, which decreases volume fraction of the ECS,
- tumors, including gliomas, where peritumoral edema can increase the volume fraction in the ECS, and increased cellularity and tortuosity significantly increase the diffusion pathways (32), and
- vasogenic edema may cause astrocytes to swell and decrease ECS volume.

While diffusion is movement of substances along a concentration gradient from areas of high concentration to areas of lower concentration, bulk flow is the movement of water and solutes along a pressure gradient. Notably, endogenous bulk flow of interstitial fluid is largely not understood. For example, it is assumed that size of the substance matters when determining extent of bulk flow, as is true for diffusion. However, when efflux of molecules with different molecular weights were measured, all cleared with the same rate constant (32). Under normal conditions, bulk flow is thought to be primarily restricted to the perivascular or Virchow–Robin space surrounding capillaries, rather than existing throughout the interstitial space (32). How this changes in the face of increased intratumoral or intracranial pressure or with administration of substances under low continuous pressure is currently under investigation.

## Circumventing Barriers to the CNS for Drug Delivery

Over the past two decades, clinical applications and investigations of cytotoxic agents, molecularly targeted agents, biologic response modifiers, and immunomodulatory agents have increased for a number of diseases, including CNS tumors. Along with this have come investigations into manipulating the BBB. Radiation therapy, osmotic disruption, focused ultrasound, and pharmacologic manipulation by bradykinin and its agonists are each associated with increased permeability of the BBB, but their effects are non-specific and time limited (34). Conversely, glucocorticoids and bevacizumab are thought to stabilize or decrease permeability of the BBB (35, 36). Attempts to bypass the CNS barriers and optimize delivery to their sites of action have been explored using alternative administration techniques; those that may be applicable to DIPG are listed in Table 3. Each has advantages and disadvantages.

**Table 3:** Techniques to overcome the blood:brain barrier (BBB).

Technique	PK advantage	Disadvantage
High-dose systemic chemotherapy	Higher *C*_max_ in circulation may result in higher *C*_max_ in central nervous system (CNS) (assuming linear increase in BBB penetration)	Toxicity if threshold for BBB penetration is reached, toxicity is increased without increasing chance of benefit If no drug penetrates at low dose, unlikely to achieve drug penetration at higher doses
BBB disruption	Temporary increase in BBB penetration into CNS	Toxicity Not tumor specific Unknown exposure (adequate concentration over adequate time periods)
Inhibition of drug efflux transporters	Block drug efflux from BBB allowing increased CNS penetration	Toxicity Results in increased plasma drug levels due to decreased drug clearance (P-glycoprotein inhibitors not specific for BBB)
Intraarterial delivery	Higher drug concentrations in region supplied by artery ONLY during first pass through tumor	Streaming effect, inhomogeneous delivery (toxicity, insufficient delivery) Unable to reach tumor cells outside of area supplied by artery Once drug enters systemic circulation, no longer any PK advantage
Convection-enhanced delivery	Bypasses BBB; direct installation into tumor bed	Invasive procedure Difficult to reach all tumor cells Need to ensure adequate exposure of active agent for a long enough period of time but difficult to evaluate PK

### High-Dose Systemic Therapy

Historically, oncologists primarily employed cytotoxic agents with steep dose–response curves, with the primary treatment goal being dose intensity (the highest dose over the shortest time interval possible). Attempts to overcome limited CNS penetration of some agents using high-dose systemic delivery have been investigated (17, 18, 37, 38). The major pharmacokinetic advantage assumes a near linear increase in CNS penetration with increasing blood levels (i.e., the same *fraction* of free, unbound drug crosses the BBB), therefore increasing the systemic dose increases blood concentrations, which result in increased concentrations in CNS. This assumption is at least partially true for drugs able to cross the BBB by simple diffusion or for those where specific transporter/carrier thresholds are not reached. The result for these agents is more uniform distribution throughout the neuraxis, independent of rate or direction of CSF flow, compared with intrathecal administration or local administration, and better penetration into deep perivascular spaces and brain parenchyma. The primary disadvantages are the significant systemic toxicities, limited appropriate drugs, and the limited diseases where drugs were efficacious. This approach has been relatively successful for agents such as methotrexate and cytarabine in the treatment of CNS lymphomas and leukemia (39), but its use for children with CNS tumors remains limited primarily due to the extensive toxicity and lack of significant benefit for most primary pediatric CNS tumors. No systemically administered high-dose chemotherapy has demonstrated efficacy for children with DIPG.

### BBB Disruption

The BBB can be disrupted in a number of ways; perhaps the technique with the most clinical experience is infusion of a hyperosmotic solution such as mannitol, which has been primarily studied in adults with supratentorial malignant gliomas. While hyperosmotic infusions with mannitol cause endothelial cell shrinkage and transient opening of tight junctions resulting in increased BBB permeability of both small and large molecules (40), the major disadvantage is enhanced CNS toxicity as normal brain is also affected (41).

Other investigated techniques include administration of neuroimmune modulators, including cytokines, which can modify BBB function, integrity, transporters, and permeability; by administration of vasoactive substances such as bradykinin or bradykinin agonists (12, 42); and by focused ultrasound. For children with brainstem gliomas, BBB disruption using the bradykinin agonist, lobradamil, was investigated with concurrent carboplatin (21), and with radiation therapy and carboplatin (20). In the radiation therapy plus carboplatin study, median survival (*n* = 13) was approximately 11 months; in the phase II study of lobradamil and carboplatin, no objective responses were observed in the brainstem cohort (*n* = 12). These studies emphasize that agent selection is key: increased delivery of an inactive agent is futile. Active agents against DIPG should be identified preclinically and their characteristics evaluated for CNS penetration. BBB disruption may result in little or no increase in small lipid soluble molecules (43) or it is unable to overcome the robust efflux effect of major transporters such as Pgp (44).

### Direct Inhibition of Efflux Transporters

P-glycoprotein is a transmembrane drug efflux pump located at the BBB as well as in a variety of normal tissues, including the epithelial surface of the choroid plexus, liver, kidney, and intestines, and is expressed on some resistant cancer cells (45). Several drugs, including cyclosporine A, can inhibit Pgp. However, the inhibition is not specific for Pgp on the BBB; several studies have demonstrated that elimination of drug from the bloodstream is affected by Pgp inhibition due to inhibition of Pgp on the kidney and liver. In addition, there is concern for neurotoxicity when inhibiting Pgp at the BBB. A clinical trial was performed administering Cyclosporine A as a Pgp inhibitor, along with etoposide and vincristine to children with diffuse intrinsic brainstem gliomas (19). Notably, significant dose-limiting neurotoxicity, including seizures with white matter changes and altered consciousness with bulbar signs, mandated early closure of this study. Median survival (*n* = 7) was 11 months.

### Regional Therapy

Regional drug delivery bypasses the BBB by delivering an agent directly to its site of action, and thereby potentially increasing its therapeutic index by decreasing systemic toxicity. The pharmacokinetic advantage from *any* regional therapy results from the first pass through the target site, as once the drug enters the systemic circulation, it follows intravenous distribution pharmacokinetics (46).

#### Intraarterial (IA) Administration

Intraarterial administration of agents aims to bypass the BBB and increase intratumoral drug exposure and has been performed for more than five decades. Higher drug concentrations, and hence, increased exposure can be achieved in a specific region supplied by the artery (46, 47). Despite this, its utility and efficacy in malignant gliomas is uncertain, likely due to the limited pharmacokinetic advantage. In general, the pharmacokinetic advantage from IA administration comes from the first pass extraction of the agent from the bloodstream into the area being supplied by the selected artery (presumably to the tumor bed); once the agent re-enters the general circulation, it is redistributed as any systemically administered drug, i.e., *via* intravenous distribution (48). Hence, the best drugs in which to use this technique are those drugs delivered in their active form, with high systemic clearance (rapidly cleared from the circulation upon first pass in the liver or kidney) that otherwise penetrate into the CNS well so as to enter the brain tumor parenchyma from the arterial circulation, i.e., small, lipophilic substances. Examples include the nitrosoureas. Platinum compounds such as carboplatin have also been used, although CNS penetration is limited (<3%) and clearance is relatively low (49), making this a non-ideal candidate. In addition, the IA procedure is commonly performed while the patient is on steroids, which may decrease penetration of carboplatin agents into the CNS even further by stabilizing the BBB and BTB.

Intracarotid (and other IA) delivery of agents to the brain is directly affected by cerebral blood flow, with low blood flow allowing greater extraction per unit time (48). Potential disadvantages of IA administration include focal neurotoxicity such as retinal damage, streaming of drug within the blood vessel resulting in non-uniform mixing, risk of embolism, and hemorrhage. Despite the application of arterial delivery of agents for glioblastoma over four decades, IA delivery has had little impact on improving outcomes.

In determining pharmacokinetic parameters after IA delivery, several assumptions are made, including uniform mixing of the agent in blood, constant blood flow to the region of interest, and homogeneous distribution within the region supplied by the artery (50). With the development of microcatheters, IA delivery with distal cannulation of super-selective arteries is now possible; although this may reduce neurologic complications, this comes at the cost of increased streaming effects (48). In addition, drug delivery is limited to a more precise region, which may not reach the invasive malignant cells at the tumor edge, and only a small percentage (6%) of patients with glioblastoma have a single vessel identified as supplying the tumor (51). Because most adult malignant gliomas are supratentorial, intracarotid arterial administration techniques can be used. Although technically feasible, the only phase III study published to date demonstrated no significant differences in time to progression or overall outcome of adults with newly diagnosed glioblastoma treated with intravenous versus IA ACNU (52).

In pediatrics, approximately 50% of tumors are located in the posterior fossa. The blood supply for those located in the brainstem comes from the vertebral arteries, which converge to form the basilar artery. This is different from other blood vessels in the body which diverge and has implications regarding flow and mixing patterns. *In vitro* studies have demonstrated a significant streaming effect with intravertebral artery administration, indicating unique flow properties that result in heterogeneous drug distribution in the pontine vessels (53). This streaming can result in significant toxicities to the brainstem and cervical spinal cord in areas that receive extremely high drug concentrations, and no effect in areas that receive insufficient drug (54, 55). As with other IA techniques, any pharmacokinetic benefit would come from the first pass effect and if areas of the tumor do not receive adequate drug exposure during the initial infusion, any potential drug activity is lost.

Despite the concern for poor mixing and streaming, intravertebral arterial administration has been utilized for brainstem tumors (56), although feasibility, safety, and efficacy data are limited. In the single publication on intervertebral artery delivery of agents for brainstem tumors in humans, four patients, ages 5–59 years, received intravertebral ACNU alone (*n* = 1), with cisplatin (*n* = 2) or carboplatin only (*n* = 1). Three of the patients had astrocytoma Grade 2; one had astrocytoma Grade 3. Survival from diagnosis ranged from 14 to 36 months. The patients tolerated the therapy with the exception of transient nausea and emesis. Although tolerated, it is unclear whether there was a survival benefit given the low-grade histology and varied ages of the patients (i.e., these did not appear to be typical DIPG). To date, no clinical trial has been published demonstrating safety, tolerability or efficacy of intravertebral drug delivery for children with DIPG.

Given that the BBB still limits delivery of agents administered intraarterially (57), combination therapy with a BBB disrupting technique has been explored. While significant response rates are reported in adults with supratentorial tumors, there are only anecdotal long-term survivors after intracarotid chemotherapy with BBB disruption suggesting limited brain distribution particularly to the invasive edge, or tumor resistance. Toxicities including seizures and local toxicity can be significant. There is no literature describing this technique in children with DIPG.

#### Direct Intratumoral Placement

Direct placement of chemotherapeutic agents at the tumor site can be performed by direct injection in the tumor bed at the time of resection, or placement of extended release wafers, gels, or beads. In each case, the pharmacological advantage is circumvention of the BBB; drug distribution and dispersion then follow the principles of diffusion. Because feasibility of placement of biodegradable polymers has not yet been demonstrated for DIPG, this will not be further discussed here, although remains a potential novel area of research, particularly with nanoparticles.

#### Convection-Enhanced Delivery (CED)

Convection-enhanced delivery is a technique that bypasses the BBB by infusing agents directly into tissues utilizing a small hydrostatic pressure gradient to aid distribution of infusate in tissues (58). Normally, when substances are administered in tissues, they distribute by simple diffusion, moving from areas of high concentration to areas of lower concentration. In CED, infusate distribution primarily relies on bulk flow (i.e., a pressure gradient rather than a concentration gradient); the result is increased volume of distribution and more homogeneous distribution in the interstitial space (59).

Surrogate imaging tracers can be administered with drug infusate during CED to track distribution of an agent in real time. This is critical because, although CED properties have been established in normal brain tissues, tumor characteristics such as cystic areas or increased intratumoral pressure may affect drug distribution. As with other agents that enter the CNS, distribution by diffusion is limited to only a few millimeters for most substances; it is therefore critical that CED administration covers much, if not all, of the tumor, as drug will distribute by diffusion once the pressure gradient is relieved. This represents a crucial issue for children with DIPG, as many of these children have tumor spread outside the MRI-defined tumor area (60–62).

## CNS Pharmacokinetics—Drug Selection and Measurement

Core neuropharmacokinetic parameters that are useful to accurately and quantitatively assess BBB penetration properties of drugs include the ratio of total brain:total plasma concentrations (logBB), which can identify highly lipophilic agents with low hydrogen-bonding potential; the amount of unbound drug in brain:unbound drug in plasma; and the cell partitioning coefficients/blood:brain partition coefficient/octanol-water partition. The overall binding and distribution of unbound drug in the brain is best correlated with and described by the octanol-water partitioning coefficient (16); higher ClogP corresponds to higher volumes of distribution of unbound drug in brain. ClogP values greater than 1 mL × g brain^−1^ indicate intracellular accumulation and/or excessive brain tissue binding as this value exceeds total brain water volume (which is 0.8 mL × g brain^−1^) (16, 63). When using ClogP to assess brain exposure, it must be kept in mind that it does not account for transport; substances with a large volume of distribution or a short-half life will have lower concentrations, exposure, and residence time at the CNS tumor site (12). In order to improve brain exposure, polarity, and/or hydrogen-bonding capacity of the agent must be decreased (16).

### CSF Measurement and Its Relationship to Brain

Because the CSF is in contact with the interstitial fluid, it provides the most accessible, relatively non-invasive, means to assess drug delivery to the brain. In contrast to brain tissue sampling in small animal models, it also allows for obtaining multiple samples over time to better assess pharmacokinetic parameters. However, CSF is a surrogate of CNS tissue penetration, and like all models, assumptions are made. Certain pharmacokinetic principles should be emphasized:

- Most pharmacokinetic models use concentration of a drug over time (exposure) in a body compartment, i.e., partitions such as plasma, CSF, and tissue, in which the drug is assumed to be well mixed. As noted above, this may not be true for CSF.
- In the absence of transport mechanisms, the exchange of free (unbound) drug between plasma and CSF should have the same rate constant in both directions at steady state (11). Without CSF turnover, free drug concentration at steady state would be equal in CSF and plasma.
- Because CSF *does* turnover [rate of CSF production is 30 cm^3^ h^−1^ in humans (64)], there is an inherent lower concentration of drug in CSF than plasma. Therefore, there is a physiological sink in addition to the BBB when drug concentrations are measured in CSF over time (11). A low CSF concentration may underestimate the concentration in tissue parenchyma. Concentration of drug measured in the CSF depends on both time and location of sampling due to this sink (64).

### ECF Measurement

Microdialysis is considered the gold standard for determination of local substance concentration in a tissue or extracellular fluid (Figure 3). In MD modeling, it is assumed that substances distribute in tissues exclusively by diffusion through the ECS (65). The MD probe must be small enough to stay in the ECS, non-toxic, and not affect interstitial osmolarity. A drug’s *in vivo* concentration in interstitial space is inferred based on measurement of the drug concentration in a dialysate. *In vivo* MD studies must take into account the tissue diffusion properties to meaningfully interpret quantitative MD data because the MD probe creates a concentration gradient and can limit access to the probe. Because tissue metabolism, diffusion, and microvascular exchange affect probe behavior, *in vitro* probe calibrations have limited applicability. Therefore, recovery of the probe must be determined in the tissue as recovery in a free solution will not be applicable (32, 65). However, many published studies of MD in humans with CNS tumors utilize *in vitro* calibration techniques and, hence, do not consider tissue characteristics, limiting their interpretation and usefulness.

### Measuring Drug Concentration in Tissue

*Ex vivo* techniques, such as measuring drug in a brain homogenate or in brain slices, typically assesses the concentration of the unbound fraction of drug after a single dose or at steady state. Using this technique, the volume of unbound drug distribution in the brain can be determined, but *only at a single timepoint* and may not allow for identification of loco-regional differences (66). Several issues must be considered when assessing a drug’s concentration in brain tissue using these techniques. After drug administration, the drug (or metabolite) diffuses into the ECS at a certain concentration but may not enter the intracellular compartment. However, if tissue is homogenized, both intracellular and extracellular drug is measured and the concentration of drug per unit volume of tissue is different from concentration in ECS. Importantly, it is the concentration experienced by the cell surface adjacent to the ECS that is the effective concentration for receptor binding (32). The concentrations measured in the ECS and in tissue homogenate are related by the volume fraction, which, in normal adult brain tissue, is 15–30% (32), but again, this volume fraction is affected by cellularity and edema, and likely varies significantly and variably when tumor is present. Additional determinants of tissue concentration in brain tissue include the rate of drug exchange across capillaries and the rate of diffusion in surrounding tissue. Following local (regional) dosing of an agent, knowledge of drug transport and reaction in the tissue is necessary to predict the local concentration of the drug. Substances can be lost from the ECS by movement across the BBB, entry into cells, irreversibly binding to cell surface receptors or transporters, and by enzymatic degradation (32).

## Conclusion

Over the past decade, we have made incredible advances in our establishment of DIPG as a distinct disease entity, our understanding of its pathophysiology, and identification of disease-specific potentially active agents. We must now focus on, and incorporate, CNS pharmacokinetics in order to translate these advances into therapeutic benefit to our patients. It does not benefit the patient to identify an active drug that does not reach the tumor. It does not benefit the patient to have them undergo complex procedures for little or no pharmacokinetic benefit. Studies evaluating CNS penetration of agents being delivered for CNS tumors must take into consideration more than the blood–brain interface; more complete CNS pharmacokinetics should be evaluated. Issues such as intratumoral heterogeneity, intrapatient heterogeneity, geographic variability within the CNS, and potential for neurotoxicity need to be evaluated. Ensuring adequate delivery to the tumor site and adequate exposure of unbound drug to all tumor cells in a safe manner is the next frontier in this trek to optimizing the success of clinical trials for DIPG and improving outcomes for these children.

## Author Contributions

KW: conceived and wrote entire article.

## Conflict of Interest Statement

The author declares that the research was conducted in the absence of any commercial or financial relationships that could be construed as a potential conflict of interest.
